# Predictive Mapping of Human Risk for West Nile Virus (WNV) Based on Environmental and Socioeconomic Factors

**DOI:** 10.1371/journal.pone.0023280

**Published:** 2011-08-10

**Authors:** Ilia Rochlin, David Turbow, Frank Gomez, Dominick V. Ninivaggi, Scott R. Campbell

**Affiliations:** 1 Suffolk County Vector Control, Yaphank, New York, United States of America; 2 College of Health Sciences, Trident University International (TUI University), Cypress, California, United States of America; 3 Suffolk County Arthropod-Borne Disease Laboratory, Yaphank, New York, United States of America; University of California, Berkeley, United States of America

## Abstract

A West Nile virus (WNV) human risk map was developed for Suffolk County, New York utilizing a case-control approach to explore the association between the risk of vector-borne WNV and habitat, landscape, virus activity, and socioeconomic variables derived from publically available datasets. Results of logistic regression modeling for the time period between 2000 and 2004 revealed that higher proportion of population with college education, increased habitat fragmentation, and proximity to WNV positive mosquito pools were strongly associated with WNV human risk. Similar to previous investigations from north-central US, this study identified middle class suburban neighborhoods as the areas with the highest WNV human risk. These results contrast with similar studies from the southern and western US, where the highest WNV risk was associated with low income areas. This discrepancy may be due to regional differences in vector ecology, urban environment, or human behavior. Geographic Information Systems (GIS) analytical tools were used to integrate the risk factors in the 2000–2004 logistic regression model generating WNV human risk map. In 2005–2010, 41 out of 46 (89%) of WNV human cases occurred either inside of (30 cases) or in close proximity (11 cases) to the WNV high risk areas predicted by the 2000–2004 model. The novel approach employed by this study may be implemented by other municipal, local, or state public health agencies to improve geographic risk estimates for vector-borne diseases based on a small number of acute human cases.

## Introduction

Since its emergence in 1999, West Nile virus (WNV) triggered the largest recorded arbovirus outbreak in North America [1], [2]. Although most clinical WNV infections are either asymptomatic or flu-like, the rare (<1%) neuroinvasive disease represents the most common form of viral encephalitis in the US [3] with the fatality rate of 10% and long-term morbidity in 50% of the patents [1]. Consequently, WNV is expected to remain one of the most important mosquito-borne diseases in North America [2], [3].

In the absence of a human vaccine, vector surveillance and control are the most effective tools for arboviral disease prevention on the population level [4]. However, these programs typically have low priority and are inadequately funded [5]. Predictive geographic models of elevated arbovirus transmission risk on a sub-county level could greatly improve the use of these limited resources and lead to improved understanding of arbovirus epidemiology, ecology, and risk factors crucial for efficient detection and targeted control [6]. Accordingly, this study's objective was to develop a predictive spatial model for WNV human risk for a large suburban county using tools that are readily available to state or local public health agencies.

Vector-borne disease modeling has emerged as a methodology. Vectors and pathogen reservoirs are often associated with environmental factors [7], distinct landscape features, and ecological settings where vector, host, and pathogen intersect within a permissive climate [8]. A number of studies have attempted to link WNV human transmission risk with such environmental and landscape elements. In large cities, WNV risk factors included higher amount of vegetation, habitat fragmentation or clumpiness, open or grassy areas, poor drainage, and open water [9]–[11]. In suburban or rural areas, WNV risk factors included high population and road density, agricultural or grass areas, wetlands, open water, and streams [12]–[14]. WNV positive birds and mosquitoes can also be important environmental predictors of WNV human risk [11], [13], [15], [16]. Spatial patterns of disease risk may also be associated with socioeconomic factors due to effects of urbanization on the natural environment [17], [18]. While the data on socioeconomic WNV risk factors are still insufficient, pioneering studies in Chicago and Detroit established a positive link between neighborhoods characterized by older housing and aging white populations with increased risk for WNV infection [11], [17]. Conversely, in southern US and California, higher risk of WNV infection was associated with low income areas [18]–[20].

Many spatial analytic studies of WNV risk predictors utilized aggregated data based on administrative divisions such as census tracts or zip codes. The statistical power of analysis associated with this approach may be low due to greatly reduced sample size. It may thus be difficult to detect significant differences in disease risk where municipalities exhibit sporadic or highly clustered WNV human cases. Additionally, aggregated spatial scale characterization can be susceptible to ecological fallacy, lack of precision, and measurement error [21]. To address this issue, researchers have stated that aggregate level studies should be supplemented by individual level data [22], and requiring more spatially explicit data collection and analysis through the use of geographic information systems (GIS) [7].

Our goal was to employ vector biology and knowledge of environmental and socioeconomic risk factors to predict spatial patterns of human West Nile Virus risk in Suffolk County, New York, USA on a local scale. A large number of factors relevant to vector, host, and human ecology were tested and the significant predictors used to generate a logistic model rendered geographically by GIS tools into a county-wide WNV human risk map. Similar approach can be easily adopted by state, county, or municipal public health agencies to investigate factors associated with WNV human transmission to enhance surveillance and control efforts, and to better understand WNV landscape epidemiology.

## Materials and Methods

### Study Area

**Figure 1:**
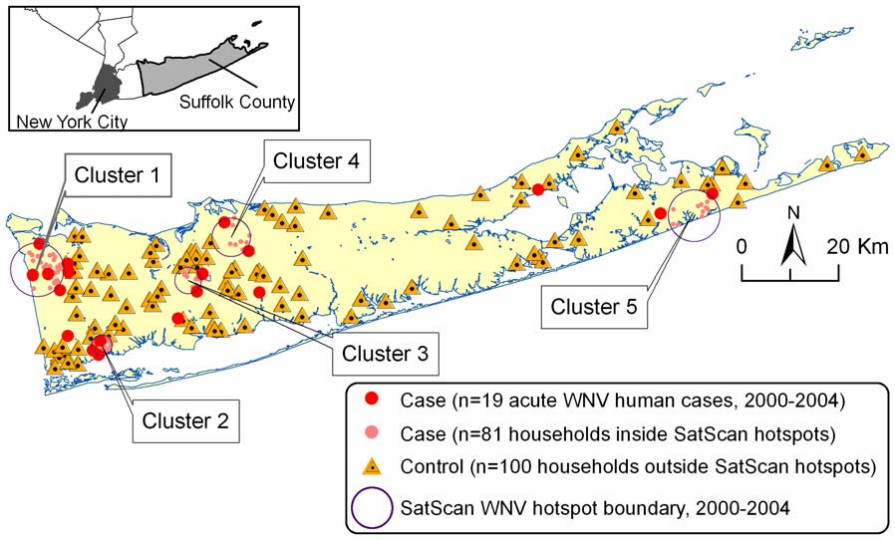
SatScan West Nile Virus (WNV) hotspot analysis and case/control selection. WNV spatial clusters (i.e. hotspots) were determined based on 19 acute WNV human cases in 2000–2004. Only Cluster 1 was statistically significant at *p*<0.05. Additional cases (n = 81) were selected inside each hotspot weighted for the number of acute WNV human cases as follows (see Table 2): Cluster 1 (n = 33), Cluster 2 (n = 17), Cluster 3 (n = 11), Cluster 4 (n = 10), and Cluster 5 (n = 10). Control household locations (n = 100) were randomly selected from outside of the WNV hotspots.

The study was conducted in Suffolk County, NY located on Long Island east of New York City (Figure 1). Suffolk County has ∼1.4 million residents and a land area of about 912 sq. miles (∼2,363 km^2^) with densely populated suburban areas, commercial and light industrial sites, agricultural areas, forested parkland, and numerous fresh and saltwater wetlands. WNV enzootic activity in birds and mosquitoes has been detected every year since the original virus introduction to North America in 1999, with sporadic epidemic outbreaks resulting in human cases.

### Study Design Overview

This study utilized a case-control design with household geographic location as the unit of analysis. Study cases included a) 19 households with acute WNV human cases in 2000–2004, and b) 81 additional households with no acute WNV human cases, but located inside the WNV hotspots delineated by SatScan spatial scan statistic [23] based on the 19 acute WNV human cases (Figure 1). Study controls included 100 households located outside of all WNV hotspot areas. Eighty one case and 100 control households were randomly selected from a geodatabase containing all Suffolk County households using ArcMap 9.1 (ESRI Inc., Redlands, CA). The sample size was predetermined by a pilot experiment using α = 0.05, π = 0.8, and the target effect size OR (odds ratio) = 2.0 [24]–[26].

The predictors of risk of WNV human transmission were derived from those reported in the literature (reviewed in the Introduction section) and developed from publicly available databases. These predictors characterized landscape elements relevant to vector and host ecology (i.e. land cover, natural and manmade wetlands, soils, habitat fragmentation), socioeconomic conditions relevant to human ecology (education, income, race, housing), and indicators of WNV activity (WNV positive mosquito pools and birds). The risk factors were evaluated at three spatial scales based on the flight range of mosquito vectors, which are the main force driving the pathogen transmission [27]. Spatial scales of up to 2.0 km radius were commonly employed in WNV epidemiological assessments [16], [28]–[32] including in Suffolk County [33], roughly corresponding to the flight ranges of important WNV vector species [34]. This study used similar spatial extents of buffering around each case and control location at 0.5, 1.0, and 2.0 km.

To evaluate the logistic model, the acute WNV human case dataset was split into two: 19 acute WNV cases in 2000–2004 were used as a training dataset for model development, whereas 46 acute WNV cases in 2005–2010 were used as a validation dataset.

### Data Sources

Human WNV infection is a reportable disease in New York State. For each acute WNV human case, Suffolk County Department of Health Services collected relevant epidemiological information including travel history. For privacy protection, the only data available for this study were the geographic locations of acute WNV human cases.

Georeferenced environmental and socioeconomic data were obtained from federal, state, and county databases (Table 1). All files were processed in ArcMap. Raster files were converted into ArcMap GRID format at 30×30 meter resolution to match the National Land Cover Database.

**Table 1:** Sources of environmental and socioeconomic data and the derived independent variables used in this study.

Source	Source URL (if available) & independent variables
**Federal Government**	
Multi-Resolution Land Characteristics Consortium	http://www.mrlc.govNational Land Cover Database (NLCD 2001): Land Use/Cover, Tree Canopy, Urban Imperviousness
NASA	http://glcf.umiacs.umd.edu/index.shtmlModerate Resolution Imaging Spectroradiometer (MODIS): Vegetation Vigor (NDVI) and Cover
United States Census Bureau (Census)	http://factfinder.census.govCensus 2000: Socioeconomic, Hydrography, Roads
United States Department of Agriculture (USDA)	http://soildatamart.nrcs.usda.govSoils (SSURGO)
United States Department of Homeland Security (FEMA)	http://www.fema.govFlood Zones
United States Geological Survey (USGS)	1999 National Hydrography Dataset (USGS NHD) through Suffolk County Information Technology Dept.
**New York State**	
Department of Environment. Conservation (NYS DEC)	http://www.nysgis.state.ny.usSuffolk County Wetlands
**Suffolk County**	
Dept. of Health Services	WNV data
Dept. of Information Technology	Hydrography (derived from1999 USGS NHD), Land Records, Groundwater retention basins
DPW Vector Control	Mosquito complaints, Mosquito larval habitat

### Data Processing and Analysis

**Figure 2:**
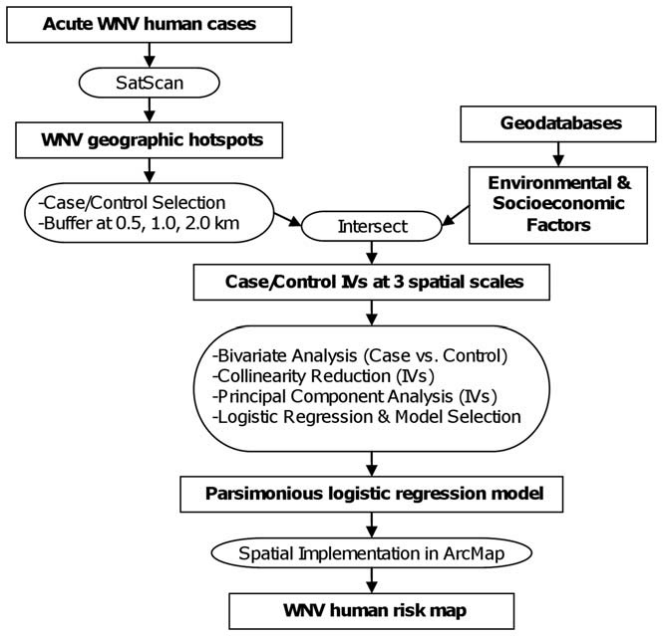
Procedure for logistic regression model construction and West Nile Virus (WNV) human risk map development. IVs - independent variables.

The data processing and analysis are schematically presented in Figure 2. The geographic extent of WNV hotspots was determined by publically available spatial cluster detection software SatScan™ [23] based on19 acute WNV human cases in 2000–2004. Purely spatial Bernoulli model included cases (i.e. 19 acute human WNV cases) and the reference grid consisting of 620 points regularly spaced at 0.02 degree interval over the entire County land area. Moving circular window with 10 km maximum radius was set to detect non-overlapping high rate clusters. Additional 81 case locations and 100 control locations were then randomly selected from within or outside the delineated clusters, respectively. These locations were buffered at 0.5, 1.0, and 2.0 km radius using ArcMap. The resulting circles were intersected with the geographic data layers containing either environmental or socioeconomic factors to extract the independent variable (IV) values at each spatial scale using ArcMap with Spatial Analyst or Hawth's Tools extensions. The output in a database format was imported into SPSS statistical software v.15.0 (SPSS Inc, Chicago, IL) for analysis.

For pairwise comparisons between cases and controls, chi-square test, t-test, 2-way ANOVA, or Mann-Whitney test were used depending on data type and distribution (normalized by transformations, if possible). To minimize collinearity, variables with Pearson's correlation coefficient >0.75 were either combined or excluded from further analysis (Table S1). Statistically significant variables (*p*<0.05) at all three spatial scales were tested for multicollinearity and either combined or removed until resolved.

**Figure 3:**
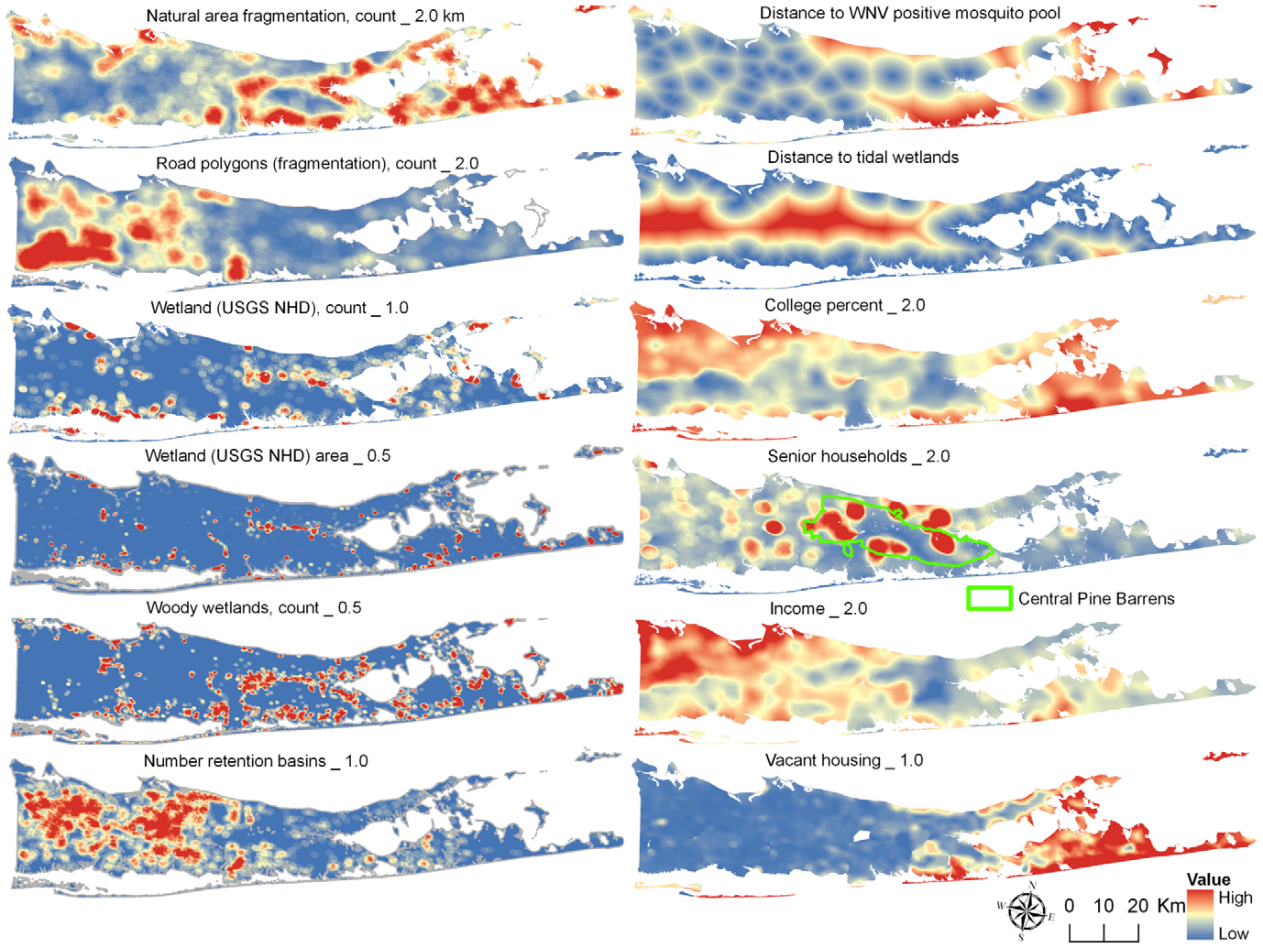
Geographic distribution of West Nile Virus (WNV) human risk predictors with the corresponding spatial scales in the final logistic regression model. Census variables were acquired at the smallest geographic unit available, i.e. census block to a block group, to census tract. The original independent variable shapefiles were converted to rasters in ArcMap GRID 30×30 meter format. The resulting grid layers were processed using Neighborhood function in ArcMap Spatial Analyst extension to produce smoothed output layers in which the value of each grid cell was a function of the cells within specified neighborhoods, i.e. the corresponding spatial scales of 0.5, 1.0, or 2.0 km (indicated by a number after each independent variable on the map). Similarly, each cell in distance grid layers (indicated as Distance to on the map) was assigned a value representing Euclidean distance to the nearest source cell.

The resulting set of IVs was characterized by Principal Component Analysis (PCA) to uncover grouping among IVs and to understand the data structure (Table S1). The entire set of IVs was then entered in a logistic regression and significant IVs at *p*<0.1 were used for the final parsimonious model with the lowest Akaike's Information Criterion (AIC). The statistical power analysis of the final model was performed using the algorithm specifically developed for multiple logistic regressions [35] and implemented in PASS 2008 software (NCSS, Kaysville, UT). Potentially serious violations of non-spatial regression assumptions by presence of spatial autocorrelation may lead to an effective reduction in sample size and increased type I error [36]. Therefore, a diagnostic test for spatial non-randomness of residuals in the final model was performed by using Moran's *I* statistics in ArcMap [37], [38]. After ascertaining the lack of global spatial clustering of the final multivariate model's residuals, the original IVs shapefiles were converted to rasters in ArcMap GRID 30×30 meter format to match the image resolution of the National Land Cover Database satellite data. The resulting grid layers were processed using Neighborhood function in ArcMap Spatial Analyst extension to produce smoothed output layers in which the value of each grid cell was a function of the cells within specified neighborhoods, i.e. the corresponding spatial scales of 0.5, 1.0, or 2.0 km for each independent variable (Figure 3). Similarly, each cell in distance grid layers was assigned a value representing Euclidean distance to the nearest source cell. The outputs were mathematically combined by Raster Calculator function in ArcMap Spatial Analyst extension using the regression coefficients in the final logistic model formula. To obtain the probability of WNV risk from the logit values (Y), the inverse logistic transformation was applied, *P*_WNV_ = e^Y^/(e^Y^+1), where *e* is a base of the natural logarithm. The final model and map were validated by 46 acute WNV cases in 2005–2010.

## Results

Initially, 64 environmental and socioeconomic factors were developed from the geodatabases listed in Table 1. Census variables were acquired at the smallest geographic unit available, i.e. census block, or, if not available, at a block group or census tract. All forest and development land use/cover (LUC) types were merged into Forest and Developed areas, respectively. A new composite variable, Natural area, contained forest, shrub, and all wetland LUC. Habitat fragmentation was operationalized as the number (or count) of separate Forest, Wetland, or Natural area polygons within each spatial scale. Grassy, herbaceous, barren, and agricultural LUCs were also combined (i.e. Grass). Four variables were eliminated due to high correlation >0.75: number of housing units, stream length (USGS NHD), “% Black”, and “% Other race”. Different “highest education level” categories were correlated at school and postsecondary levels, and thus combined into 2 new variables, school (some school and high school) and college (some college, college, and graduate) percent, resulting in 57 IVs (see Tables 1 and 2).

**Table 2:** SatScan™ WNV human cluster analysis and case selection.

Cluster	Radius, km	*p*	# WNV acute human cases	Weight^a^	Additional case locations^b^
1	5.5	.024	6	0.4	33
2	2.1	.407	3	0.2	17
3	2.7	.999	2	0.14	11
4	4.0	1.000	2	0.13	10
5	5.3	1.000	2	0.13	10
None^c^	NA	NA	4	NA	None

a Calculated as # WNV acute human cases/total.

b Calculated as Weight*81 (the number of cases to bring the total to 100).

c Single locations of acute human WNV cases that were not included with five spatial clusters.

NA-not applicable.

SatScan analysis of 19 acute WNV human cases in 2000–2004 identified five WNV clusters or hotspots (Figure 1). Only one cluster (Cluster 1) with 6 acute WNV human cases was statistically significant (Table 2). This cluster also contributed the greatest number of additional cases through the stratified random sampling procedure weighed for the number of actual WNV human cases in each cluster (Table 2). This case selection procedure aimed to create a set of cases that was representative of environmental and socioeconomic conditions in the vicinity of all acute WNV human cases and to minimize potential spatial autocorrelation problems. Subsequently, the 57 IVs or factors developed from the geodatabases were transformed, if appropriate, and compared pairwise between the 100 case and the 100 control locations at 3 spatial buffers (0.5, 1.0, and 2.0 km), or as a distance to the nearest feature. Thirty factors were significantly different between case and control locations at *p*<0.05 resulting in 53 independent variables due to multiple spatial scales, out of which 14 redundant IVs were eliminated to reduce multicollinearity (Table S1).

The structure of the remaining 39 IVs for 200 case and control locations was analyzed by Principal Component Analysis (PCA) using eigenvalue >1.0 to retain the principal components (PCs). A total of 8 PCs accounted for ∼73% of the total variance and were interpreted as follows (Table S1). PC1 (Urbanized/WNV) accounted for ∼22% of the total variance correlating positively with urbanization (e.g. development, roads, housing age, retention basins) and WNV activity (i.e. proximity and density of WNV positive birds and proximity to WNV positive mosquito pools), but negatively so with natural vegetation (e.g. forest). PC2 (Larval Hydrology) accounted for ∼14% of the total variance correlating positively with mosquito larval sites, wetlands, and poor drainage soils. PC3 (Affluence) accounted for ∼8% of the total variance correlating positively with income and college/graduate education and negatively so with school education (i.e. some school and high school combined). Each PC4–PC8 accounted for ∼5–7% of the total variance containing one factor type, i.e. wetlands (woody, emergent, open water), senior households, and density of WNV mosquito pools. Four variables with loadings on more than one PC were omitted from PCA (Table S1).

The same 39 IVs were entered in a logistic regression model. The full model classified correctly (i.e. as cases or controls, respectively) 93% of the cases including 16 out of 19 (84%) acute WNV human cases in 2000–2004, and 91% of the controls with a U-shape distribution around the cut value of 0.5. Out of 39 original IVs in the full model, 14 IVs were statistically significant at *p*<0.1 and were used for the final model created by removing the least significant IV and introducing a new IV with the goal of increasing the parsimony while preserving the overall model fit. The final reduced model contained 12 IVs classifying correctly 89% of the cases including 16 out of 19 (84%) acute WNV human cases in 2000–2004 (identical to those in the full model), and 85% of controls (Table 3). Further exclusion of all non-significant variables at α = 0.05 resulted in a model with fewer predictors (n = 9), but also with significantly reduced sensitivity and the overall fit of the model (data not shown; see [39] for details). There was no statistical difference between the full and the final models with 12 IVs (−2LL ratio test, *p* = 0.181), however, the final model (AIC = 147.5) was more parsimonious than the full model (AIC = 168.1). Given the sample size n = 200, statistical significance α = 0.05, power π = 0.8, the baseline probability of the high WNV risk (*P*_WNV_ = 0.5–1.0) P0 = 0.3, and the multiple correlation coefficient R^2^ = 0.53 estimated for the model as an average of (1 - tolerance) for all IVs, the final model's effect size was OR = 1.9 which was in line with the original goal.

**Table 3:** Final logistic regression model for WNV human risk.

Variable	Spatial scale	Std B^b^	B	S.E.	Wald	*P*	Exp(B)	95.0% C.I.	
College education, percent	2.0 km	2.56	0.307	0.073	17.6	<.001	1.36	1.18	1.57
Distance to tidal wetland, ft	NA	1.51	0.037	0.010	14.2	<.001	1.04	1.02	1.06
# senior households, age>65	2.0 km	−1.21	−1.594	0.481	11.0	.001	0.20	0.08	0.52
Distance to WNV positive mosquito pool (2000–2004), ft	NA	−1.29	−0.034	0.010	10.8	.001	0.97	0.95	0.99
Woody wetlands, count	0.5 km	−2.31	−1.228	0.379	10.5	.001	0.29	0.14	0.62
Road polygons (fragmentation), count	2.0 km	1.01	0.222	0.103	4.6	.031	1.25	1.02	1.53
Median household income, $	2.0 km	−0.98	−6.3×10^−5^	0.0	4.5	.033	1.00	>0.99	<1.00
Wetland (USGS NHD)^a^ area, sq. ft	0.5 km	1.96	1.3×10^−5^	0.0	4.0	.046	1.00	>1.00	<1.01
Natural areas fragmentation, count	2.0 km	0.75	0.198	0.101	3.8	.051	1.22	1.00	1.49
# retention basins	1.0 km	−0.69	−0.483	0.282	2.9	.087	0.62	0.35	1.07
Vacant housing, percent	1.0 km	−0.96	−0.052	0.032	2.6	.105	0.95	0.89	1.01
Wetland (USGS NHD)^a^, count	1.0 km	0.47	0.131	0.094	2.0	.162	1.14	0.95	1.37
Constant		−0.23	−6.963	4.052	3.0	.086			

a USGS NHD- United States Geological Survey National Hydrology Dataset (1999).

b Standardized B calculated with all variables converted to their respective Z-scores.

NA-not applicable.

The spatial implementation of this model in ArcMap was used to produce the county-wide WNV human risk map.

Spatial autocorrelation in the residuals was non-significant (Moran's *I* statistics 0.092, Z-score = 1.78, *p* = 0.0744) indicating negligible to weak unexplained clustering on a global scale. Visual map examination showed large residuals (Anselin local Moran's *I*, Z-score>1.96) in both cases and controls scattered throughout the western part of the County (data not shown). Slightly higher number of large residuals in and around Cluster 4 (Figure 1) suggested poorer fit and less predictive power of the model in that area [40]. Given the non-significance of global spatial autocorrelation in the residuals after including all factors in the final multiple regression model, it was concluded that there was no statistical bias in the overall regression analysis [41].

**Figure 4:**
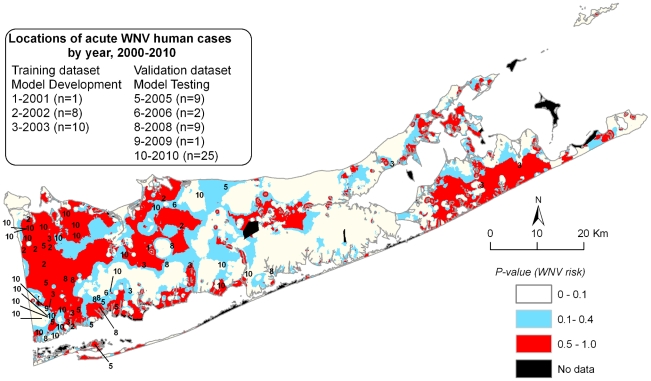
Suffolk County West Nile Virus (WNV) human risk map based on the final logistic regression model. WNV risk probabilities range from *p* = 0 (lowest) to *p* = 1.0 (highest). High WNV risk areas are defined as WNV human risk probability *p*>0.5. The geographic locations of acute WNV human cases in 2000–2004 (training dataset to generate the risk map) and 2005–2010 (validation dataset to assess the map accuracy) are shown. “No data” areas were generated due to incomplete census variables coverage in federal holdings (Plum Island, Brookhaven National Lab, parts of Fire Island National Seashore), privately owned islands, and along some sinuous coastlines.

To generate the WNV human risk map, the shapefiles of individual IVs were processed by Neighborhood function (ArcMap Spatial Analyst extension) to calculate statistics for each IV at the corresponding spatial extent of 0.5, 1.0, or 2.0 km. The resulting raster files (see Figure 3 for each factor and spatial scale combination indicated on the map) were merged by Raster Calculator function using the final logistic regression equation with minor adjustments to correct for discrepancies between tabular and raster processing. The output was transformed into a probability scale yielding the final WNV human risk map (Figure 4). Census variables lacked complete coverage in some locations such as federal holdings (Plum Island, Brookhaven National Lab, parts of Fire Island National Seashore), privately owned islands, and along sinuous coastlines resulting in “No data” areas in the final map representing ∼1.3% (∼31 km^2^) of the total land area in Suffolk County.

The final model and map were validated using 46 acute WNV human cases in 2005–2010 (Figure 4). The distance from each location to the high WNV risk area was calculated. A case was considered correct if located within 0.5 km from the nearest high WNV risk area to account for human mobility within typical suburban residential neighborhoods [42]. For training dataset (2000–2004), the risk map sensitivity varied from 100% in 2001 (1 out of 1) and 2002 (8 out of 8), to 80% in 2003 (8 out of 10). For validation dataset (2005–2010), the risk map sensitivity was 78% in 2005 (7 out of 9), 0% in 2006 (0 out of 2), 44% in 2008 (4 out of 9), 100% in 2009 (1 out of 1), and 72% in 2010 (18 out of 25). Out of 46 validation cases, only 5 occurred at a distance exceeding 1.0 km from high WNV risk areas. The overall risk map sensitivity of 89% (17 out of 19) for the experimental dataset was significantly higher compared to 65% (30 out of 46) for the verification dataset, Chi-square exact test *X^2^* = 4.0, df = 1, *p* = 0.047. Out of 65 WNV acute human cases in 2000–2010, 47 (∼72%) occurred in high WNV risk areas, which comprised ∼33% of Suffolk County's land area This distribution of WNV human cases was significantly different from random based on the land area (21 out of 65 expected), Chi-square exact test *X^2^* = 20.1, df = 1, *p*<0.001. Only 11% of all acute WNV human cases (7 out of 65) occurred at a distance exceeding 1.0 km from high risk areas delineated by the model.

## Discussion

To be useful for disease surveillance and control program, a geographic human risk model should a) use predictors that are easily available and interpretable, b) be accurate against independent data, and c) generate outputs that can assist control decisions [43]. Many of the previously reported WNV risk models [9], [12]–[14], [16] were lacking in one or more of these aspects, being too conceptual and technically complex rather than practical and easily interpretable, or providing insufficient spatial resolution for targeted control action. To overcome these problems, we developed a simple yet statistically rigorous protocol to create interpretable and testable model integrated with the county WNV surveillance and control program. In addition to providing operational county-wide WNV human risk map, the model allowed a close examination of the most significant risk factors selected from a large pool of environmental and socioeconomic parameters relevant to WNV ecology and epidemiology.

Socioeconomic conditions have emerged as the key determinants of WNV human risk [11], [17], [18]. Urbanization and increased WNV activity were linked by the number of studies [18], [44]. Similarly, in our study, WNV human risk was also associated with urbanization effects such as increased road density and fragmented natural areas, but even more so with a particular type urban environment characterized by a higher proportion of people with college education, which was the most significant risk factor in the model (Table 3). Increased percent of people with college education and median income were positively correlated with Affluence PC, but had an opposite effect on WNV human risk in the model (i.e. the former as a risk factor and the later as a protective factor) suggesting middle class suburban neighborhoods rather than higher income communities as the areas with the greatest WNV human risk. This conclusion was similar to that reached by investigators in Chicago and Detroit where the highest WNV human risk was associated with the middle class neighborhoods or “inner suburbs”, but was much lower in the more affluent high income “outer” suburbs, or in the impoverished inner cities [17].

The middle class suburban areas appeared to support the appropriate combination of vegetation, open space, and potential vector habitat favoring WNV transmission. Wealthier neighborhoods had more vegetation, more diverse land use, and less habitat fragmentation likely resulting in higher biological diversity potentially protective against the WNV human transmission, e.g. the avian host “dilution effect” [45]. Interestingly, while WNV risk appears to be the highest in the middle class suburban environment in the north-central and northeastern US ([17], this study) in southern and western US the higher risk of WNV infection was often associated with low income areas [18]–[20]. This discrepancy may have multiple explanations. One is the differences in vector ecology between these regions of the United States. In the south and west, mosquito vector populations were strongly associated with urban breeding sources such as containers [19] and abandoned swimming pools [46]. In contrast, the main enzootic and possibly epidemic vector in the northern US, *Culex pipiens*, was most prevalent in urban areas with significant vegetation cover and plentiful avian hosts [47]. However, high vector densities are not always correlated with human risk [18], [48]. Additional contributing factors may include differences in low income urban habitat, with densely built up inner cities in the northern US versus single family home with adjacent vegetation and swimming pools in southern or western US [18], [20]. Variability in patterns of human behavior may have also played a role leading to higher risk of exposure to mosquito bites in lower socioeconomic status populations in the southern and western US [18], [26].

Although these findings have clearly demonstrated the interdependence between socioeconomic and natural environments, such relationships may be multifaceted. For example, elderly population and vacant housing were negatively associated with WNV human risk in our study, contrary to the expectation [3], [10], [11], [46]. Examination of these factors' geographic distribution (Figure 3) revealed their concentration in less developed areas of the County corresponding to the unique Central Pine barrens region or the affluent and rural east end of Long Island's South Fork. Both factors had no or negative association with Urbanized/WNV PC (Table S1), and, therefore, may have represented a proxy for specific physiogeographic regions less favorable for WNV maintenance or transmission, a possibility noted in previous studies [11].

Among predictors of WNV human risk in the model, habitat fragmentation (operationalized as number of road polygons and disconnected natural areas) is an important factor facilitating transmission of many vector-borne diseases worldwide. The anthropogenic habitat fragmentation effects vary from increased erosion and surface water accumulation, reduced species richness, extinction of top predators with increase of prey species, enhanced host-vector interactions, and a shift to anthropophyllic feeding by vectors [49], [50]. Association of higher road density or habitat patchiness with elevated WNV activity or human risk was also previously established [10], [12], [51].

Habitat fragmentation by roads and WNV enzootic activity parameters (positive birds and distance to a positive mosquito pool) were grouped within Urbanized/WNV PC in our study. While proximity to a WNV positive mosquito pool was a strong human risk factor in the model as expected, WNV positive mosquito pool density was not significant in the final multiple regression model, and, moreover, was not correlated with Urbanized/WNV PC. These results supported inconsistent association of WNV positive mosquito pool density with human risk [13], and suggested proximity to a WNV positive mosquito pool as a stronger indicator of human risk. WNV positive birds, another common human risk indicator used in surveillance and risk assessment [11], [12], [15] was also not significant in our model supporting the assertion that bird-based surveillance may be unreliable due to its dependence on the general public, bias toward urban areas, and sensitivity to herd immunity [7], [21], [52], [53].

The remaining predictors in this study's model described natural or manmade wetlands. WNV human risk association with freshwater wetlands and open water, potential mosquito larval habitat, has been well documented [10], [14], [54]. In our study, freshwater wetland area and fragmentation (count) grouped with Open Water PC and Larval Hydrology PC pointing to the same link. Larger wetland areas with increased fragmentation could create aquatic edge habitat favorable for mosquito larvae and contain intermittent standing water hostile to larvivorous fish. Conversely, woody wetlands (a strong negative predictor in the model) flood in the spring and remain largely dry through the summer supporting mostly floodwater mosquitoes that are unlikely to vector WNV due to early season emergence and lower vector competence compared to *Culex* and container breeding *Aedes* species [34]. In addition, woody wetlands aggregate in more rural parts of the county exhibiting lower WNV activity in general (Figure 3). Another negative term in the model was proximity to tidal wetlands. The possible explanations for this finding may include long range migration by salt marsh mosquitoes, routine vector control activities near tidal marshes due to higher level of mosquito populations, greater use of mosquito repellents or window screens, or a modeling artifact due to the stratified sampling selecting more human cases from the inland WNV hotspots in 2000–2004. Similarly, negative association of manmade wetlands, i.e. retention basins with WNV human risk in the model can be explained by lower number of retention basins in drier areas, which nevertheless may serve as more efficient amplification foci for WNV transmission cycle by concentrating vectors and hosts in isolated vegetated sites within residential areas [48].

In addition to interpretability, predictive accuracy assessment is crucial for determining the model's utility, yet vector-borne disease model evaluation mostly focused on the past rather than predicted outcomes [8]. To address this issue, we created two independent data sets for model training and validation purposes. Although the model accuracy was significantly higher for the training dataset in 2000–2004, 89% (17 out of 19) versus 65% (30 out of 46) for the verification dataset in 2005–2010, the majority of acute human cases in the validation dataset (41 out of 46) occurred either inside or in close proximity (i.e. <1.0 km) to the WNV high risk areas delineated by the 2000–2004 model (Figure 4). There are many potential sources of error and confounding to explain reduced model sensitivity for verification dataset. Environmental and socioeconomic factors were assumed static, but may have changed over the time period covered by the model. WNV human transmission may have been affected by increased vector control activities at targeted areas with human cases as well as many coastal areas near the south shore of Long Island, which routinely experience high mosquito populations to require control. Human behavior was another potential confounder not captured by the model. For instance, it is not known whether the socioeconomic factors might be correlated with repellent use or tolerance for mosquito bites.

Apart from those limitations due to incomplete data or imperfect understanding of the epidemiological and biological processes, multiple regression analysis may encounter two important methodological caveats, namely collinearity and spatial autocorrelation. Collinearity is caused by inclusion of many highly correlated environmental and socioeconomic factors in a model leading to instability in the estimation of the partial regression coefficients. This is especially relevant to WNV risk modeling, since the complex epidemiology of the virus transmission cycle typically necessitates entering multiple factors at several spatial scales in the analysis. One plausible approach to deal with this problem is to use principal components analysis to reduce the dimensionality among the predictors [41]. Regression analysis using principal components identified in this study resulted in similar but slightly less accurate estimates compared to individual predictors (data not shown; see [39] for details). Accordingly, regression analysis with individual predictors was preferable in this case. Another important issue that may impede correct application of standard statistical tests in a geographic context is spatial autocorrelation (i.e. lack of independence) found in most natural ecological phenomena [36]. Failure to account for positive spatial autocorrelation in the model may cause test statistics to be reported as significant, when they are not due to the effectively reduced number of the degrees of freedom. In multiple regressions, spatial autocorrelation can be detected by clustered distribution of the residuals [38]. If not statistically significant, non-spatial models may be used [41]. Conversely, if significant clustering of the residuals is detected, spatial regression analysis is warranted; not allowing for spatial autocorrelation in the models has been a common source of error in epidemiological analyses [55]. Geographically Weighted Regression (GWR) function for spatial linear regression analysis is included with ArcGIS 9.3 Spatial Analyst. Only specialized software is currently available for spatial logistic regression implementation. Relatively simple methodological procedures have been reported in literature (for example [56]), and applied to improve prediction and understanding of causal factors using spatially explicit models for vector-born disease risk (for example [37], [57], [58]).

Despite limitations and technical caveats, in our study, the WNV risk map developed using 2000–2004 human cases predicted the locations of the 2005–2010 human cases with sufficient operational accuracy. The map serves in conjunction with the entomological data, which did not fully accounted for the patterns of WNV human transmission risk in Suffolk County [48]. In practical terms, the WNV human risk map may assist with selecting surveillance sites, guiding preventive control measures such as catch basin and marsh larviciding, and determining the thresholds for triggering reactive control activities, i.e. adulticiding. Such activities in the highest risk areas may commence earlier in the season or after detection of WNV enzootic activity. Targeted surveillance and control efforts prioritized for high WNV risk areas should lead to increased public health protection during outbreaks while reducing costs, labor, and environmental impacts associated with these measures. Serious WNV outbreaks in lower risk areas may suggest changes in the environment, virus biology (e.g. a new strain), or ecology (e.g. a new vector species), assisting and directing further epidemiological inquiry.

This study demonstrated the feasibility of state or local level GIS based modeling using limited epidemiological data to create risk maps for outbreak investigations, arbovirus surveillance, and scientific discovery. The risk map may be further improved by incorporating entomological and climatic data ultimately leading to a real-time risk model. However, the increased complexity of such undertaking will likely require much closer cooperation than is currently in place between local public health agencies and academic or research institutions.

## Supporting Information

Table S1 Environmental and socioeconomic independent variables in this study. Bivariate analysis compared cases (locations of acute WNV human cases and households within human WNV spatial clusters identified by SatScan) and controls (locations of households outside of SatScan WNV spatial clusters) at 3 spatial scales. Principal component analysis grouped significant independent variables determined by bivariate analysis and assisted in the interpretation.
(DOC)
