# Supplementary material for: Predictive Mapping of Human Risk for West Nile Virus (WNV) Based on Environmental and Socioeconomic Factors
Source: PLoS One. 2011 Aug 10;6(8):e23280. doi: 10.1371/journal.pone.0023280 (PMC3154328; doi:10.1371/journal.pone.0023280)
Supplement: Table S1 — Environmental and socioeconomic independent variables in this study. Bivariate analysis compared cases (locations of acute WNV human cases and households within human WNV spatial clusters identified by SatScan) and controls (locations of households outside of SatScan WNV spatial clusters) at 3 spatial scales. Principal component analysis grouped significant independent variables determined by bivariate analysis and assisted in the interpretation. (DOC) [file pone.0023280.s001.doc]

| **Environmental or socioeconomic factors** | **Variable name** | **Bivariate analysis**  **significance (sign)**^d^ | **Principal component**  **analysis (sign)**^e^ |
| --- | --- | --- | --- |
| ***Race or Ethnicity*** | | | |
| White, percent | White | N |  |
| Asian, percent | Asian | Y (+) | Urbanized/WNV (+) or Multiple PC (excluded) |
| Latino, percent | Latino | N |  |
| ***Education*** | | | |
| No school, percent | No school | N |  |
| School^a^, percent | School | Y (-) | Affluence (-) |
| College^b^, percent | College | Y (+) | Affluence (+) |
| ***Housing*** | | | |
| Housing age, years | House age | Y (+) | Urbanized/WNV (+) |
| Vacant housing, percent | House vacant | Y (-) | Urbanized/WNV (-) |
| ***Population/Income*** | | | |
| Median household income, $ | Income | Y (+) | Affluence (+) |
| Median age, years | Age | N |  |
| Total population | Population | N |  |
| # senior households, age>65 | Senior hshlds | Y (-) | Senior households (+) |
| Urban or rural category | Urban | N |  |
| ***Roads*** | | | |
| Road length, ft | Road length | Y (+) | Urbanized/WNV (+) |
| Road polygons (fragmentation), count | Road fragment | Y (+) | Urbanized/WNV (+) |
| ***Land Use/Cover*** | | | |
| Tree canopy, percent | Canopy | N |  |
| Imperviousness, percent | Imperviousness | N |  |
| Vegetation vigor (NDVI) | NDVI | N |  |
| Tree/shrub cover, percent | Tree cover | N |  |
| Open water, area | Water | Y (+) | Open water (+) |
| Shrub, area | Shrub | N |  |
| Forest, area | Forest | Y (-) | Urbanized/WNV (-) |
| -(Developed areas)^c^ | Developed | Y (-) | Urbanized/WNV (-) |
| Open grassy, barren, or agriculture, area | Grass | Y (-) | Urbanized/WNV (-) |
| Woody wetlands, area | WoodyWet | Y (-) | Woody wetlands (+) |
| Woody wetlands, count | WoodyWet cnt | Y (-) | Woody wetlands (+) |
| Emergent wetlands, area | EmergWet | N |  |
| Emergent wetlands, count | EmergWet cnt | Y (+) | Emergent wetland/Flood (+) |
| Natural areas fragmentation, count | Natural areas | Y (+) | Multiple PC (excluded) |
| ***Freshwater Wetlands*** | | | |
| Wetland (NYSDEC), area | WetDEC | N |  |
| Wetland (NYSDEC), count | WetDEC cnt | Y (+) | Larval hydrology (+) |
| Wetland (USGS NHD), area | WetUSGS | Y (-) | Open water (+) |
| Wetland (USGS NHD), count | WetUSGS cnt | Y (+/-) | Open water (+) or Larval hydrology (+) or Multiple PC (excluded) |
| Retention basins, count | Sumps | Y (+) | Urbanized/WNV (+) |
| ***Flooding*** | | | |
| Flood area (FEMA) | Flood Zone | Y (+) | Emergent wetland/Flood (+) |
| ***Streams*** | | | |
| Census Bureau hydrology length, ft | StreamCensus | N |  |
| ***Soil*** | | | |
| Hydric soil, area | Hydric soil | N |  |
| Hydric group, area | Hydric group | N |  |
| Poor drainage, area | Drainage | Y (+) | Larval hydrology (+) |
| Suitability for shallow water, area | Shallow water | N |  |
| ***Mosquito and West Nile Virus*** | | | |
| # complaints 1999-2008 | Complaint | N |  |
| # known larval sites | Larval site | Y (+) | Larval hydrology (+) |
| # WNV positive birds, 1999-2004 | WNV bird | Y (+) | Urbanized/WNV (+) |
| # WNV positive mosquito pools, 2000-2004 | WNV mos pool | Y (+) | WNV mosquito (+) |
| ***Distance to the nearest feature, ft*** | | | |
| Open water | Water_Dis | N |  |
| Wooded wetland | WoodyWet_Dis | N |  |
| Emergent wetland | EmergWet_Dis | N |  |
| Retention basin | Sump_Dis | N |  |
| Tidal wetland | TidalWet_Dis | Y (+) | Multiple PC (excluded) |
| Wetland, USGS NHD | WetUSGS_Dis | Y (-) | Larval hydrology (-) |
| Stream, Census | StreamCensus_Dis | N |  |
| Flooding zone | FloodZone_Dis | N |  |
| Sewage treatment plant | Sewage_Dis | N |  |
| Mosquito complaint | Complaint_Dis | N |  |
| Known larval site | LarvalSite_Dis | N |  |
| WNV positive bird, 1999-2004 | WNVBird_Dis | Y (-) | Urbanized/WNV (-) |
| WNV positive mosquito pool, 2000-2004 | WNVMosPool_Dis | Y (-) | Urbanized/WNV (-) |

^a^”Some school” and “High school” combined.

^b^”Some college”, “College”, and “Graduate” combined.

^c^The “Developed areas” independent variable was subjected to inverse transformation for statistical analysis.

^d^Case versus Control comparison at 3 spatial scales (0.5, 1.0, 2.0 km): Student t-test, Mann-Whitney, or 2-way ANOVA. N-no or Y-yes refer to statistical significance. The sign associated with each statistic (“+” or “-“) shows the “direction” of the effect of the variable on the case group, i.e. “+” sign indicates that the higher values of the variable are associated with case households.

^e^Principal Component Analysis (PCA): 8 PCs with eigenvalue>1.0. Some variables loaded on 2 factors (Multiple PC) and were thus omitted from PCA. The sign of the variable loading is shown. Note that ”Distance to the nearest feature” category is inversely related to proximity, so a “-“ sign indicates closer distance.
